# Creative and Critical Thinking and Modelling: Confluences and Implications for Science Teaching

**DOI:** 10.3390/jintelligence13090111

**Published:** 2025-08-31

**Authors:** Marta Gómiz-Aragón, María del Mar Aragón-Méndez, Rui Marques Vieira, Celina Tenreiro-Vieira, José María Oliva

**Affiliations:** 1 Faculty of Science Education, University of Cádiz, 11519 Puerto Real, Spain; marta.gomiz@uca.es (M.G.-A.); mariadelmar.aragon@uca.es (M.d.M.A.-M.); 2 Department of Education and Psychology, Research Centre on Didactics and Technology in the Education of Trainers, University of Aveiro, 3810-198 Aveiro, Portugal; rvieira@ua.pt (R.M.V.); cvieira@ua.pt (C.T.-V.)

**Keywords:** creative and critical thinking, modelling, argumentation, analogies, socioscientific issues

## Abstract

Contemporary society demands the development of creative critical thinking (CCT) as a fundamental objective in science education. However, there appears to be a dissonance between this recognised need and its actual implementation in educational practices. This study explores the potential of modelling practices to intentionally, explicitly, and reflectively integrate the development of CCT in educational settings. To examine this possibility while laying the foundations for future research, the theoretical frameworks of CCT and modelling are synthesised, and their possible points of convergence are analysed in order to test the proposed hypothesis. Two elements that may strengthen their synergy are identified: first, the resources for modelling, such as analogies, which activate analytical, evaluative, creative, and argumentative skills, thereby fostering critical dispositions and a deeper understanding of the nature of science. Second, argumentation, closely connected with communication and information management, is considered a relevant component, especially when addressing socioscientific issues. While further empirical research is needed, the analysis indicates that modelling practices could contribute to the development of CCT.

## 1. Introduction

Our society is characterised by complexity, volatility, and uncertainty. The growth of science, technology, and new forms of communication has opened new possibilities for addressing global crises and challenges. However, these developments have also given rise to new problems. Among them are the proliferation of misinformation—such as fake news and ideological manipulation—the emergence of pseudoscience, scientific denial, and the rejection of scientific evidence without critical reasoning.

There is no single solution to these issues. Nevertheless, all proposals highlight the need to educate citizens in social responsibility. This education should empower individuals to participate democratically and respond to emerging challenges. International organisations and educational systems have acknowledged this need. As a result, the promotion of critical thinking across different areas has been incorporated as a curricular objective, although it may not have received sufficient emphasis.

Currently, science education recognises the development of critical thinking as a fundamental goal. This goal is increasingly relevant for citizens in democratic societies shaped by science and technology (Tenreiro-Vieira and Vieira 2022). Alongside critical thinking, these and other researchers are placing growing emphasis on the promotion of creativity. Creativity is essential for developing new solutions to emerging problems and for advancing towards a more humane and sustainable society. Research in science education has generated several proposals to foster critical thinking, although fewer studies have addressed creativity. Nevertheless, a significant gap remains between the recognised importance of these competencies and their integration into classroom practice.

Given the current context, science education must take on the challenge of integrating critical and creative thinking into school science practices. This integration should be intentional, explicit, and reflective (Tenreiro-Vieira and Vieira 2022). *Intentional* refers to integration that is deliberately planned and aligned with instructional goals. *Explicit* means that critical and creative thinking are clearly addressed, taught, and made visible to students throughout science instruction. *Reflective* implies encouraging both teachers and students to engage in metacognitive processes that allow them to evaluate, question, and refine their thinking during scientific activities.

This article proposes that modelling practices have the potential to contribute to this goal. To that end, it presents a brief overview of the theoretical frameworks related to critical and creative thinking, as well as to modelling. It then analyses their points of convergence in order to systematise the inclusion of critical and creative thinking in modelling practices.

## 2. Critical Thinking

### 2.1. Conceptualization and Characterization of Critical Thinking in Science Education

Critical thinking has been studied from various disciplines, including philosophy, cognitive psychology, pedagogy, and science education (Santos 2017). However, no clear consensus exists on its precise definition or its dimensions.

Ennis (1987, 1996) defines critical thinking as rational and reflective thinking that helps decide what to believe or do. It encompasses both dispositions and cognitive skills. Scriven and Paul (2007) describe it as a disciplined intellectual process that analyses and evaluates information from different sources, such as observation, experience, or communication, to guide beliefs and actions.

Paul and Elder (2001, 2006) conceptualise critical thinking as a way of thinking that improves the quality of one’s own thought by applying intellectual standards to its structures. They also highlight the relationship between critical thinking and creativity. In any innovation process, it is necessary to evaluate the feasibility of new ideas, refine solutions, and make ethical and rational decisions. Without creativity, critical thinking is limited to scepticism. Without critical thinking, creativity lacks rational evaluation. Lipman (2003) likewise argues that creativity guides critical thinking by opening new possibilities and generating hypotheses. Prado-Arenas et al. (2022) state that creative thinking complements critical thinking by facilitating the connection of ideas and the reasoned expression of original ideas.

Johnson and Hamby (2015) warn against confusing the term “critical thinking” with reasoning, knowledge, problem-solving, decision-making, or metacognition. They emphasise the need to manage criticism of one’s own ideas. Kuhn (2021) reinforces this idea by highlighting the role of metacognition as both a skill and a disposition. He stresses that metacognition involves awareness of one’s own thinking and reflection on both one’s own and others’ thinking as objects of knowledge. Building on this, Guamanga et al. (2025) underscore the importance of critical thinking in fostering cognitive self-regulation and socio-emotional competencies.

In science education, critical thinking has garnered increasing attention. It is intrinsically connected to the very nature of scientific activity (Santos 2017). Specific content and problems within science curricula require or encourage its application (Bailin 2002). Furthermore, critical thinking can develop in parallel with scientific thinking (García-Carmona 2023).

A growing number of researchers advocate for a broader conception of critical thinking that includes not only cognitive and epistemic aspects but also values, emotions, and ethical considerations. Various taxonomies seek to characterise critical thinking and promote its development in the classroom. Jiménez-Aleixandre and Puig (2022) highlight components such as cognitive and epistemic skills, critical dispositions, independent thinking, and critical discourse analysis. Franco-Mariscal et al. (2024) propose a three-dimensional model based on knowledge, skills, and dispositions. Vila-Tura et al. (2022) include values, emotions, and the outcomes of critical thinking. Vázquez-Alonso and Manassero-Mas (2018a) identify four dimensions: reasoning, creativity, problem-solving, and judgement, the latter encompassing source evaluation, communication, and metacognition. According to García-Carmona (2023), argumentation can be based on scientific evidence or on ethical, social, economic, or emotional values, all of which are regulated by ethical considerations (Helm 2001).

In summary, critical thinking is a form of higher-order thinking. It consists of a set of capacities that can be developed through both epistemic and non-epistemic scientific practices (García-Carmona 2023). Furthermore, it enables individuals to make decisions, solve problems, and become engaged citizens committed to a more just and sustainable society (Tenreiro-Vieira and Vieira 2022; Vila-Tura et al. 2022).

### 2.2. Critical Thinking from the Science-Technology-Society Perspective

The development of critical thinking is closely linked to scientific literacy within the Science-Technology-Society (STS) framework, which promotes a functional understanding of scientific knowledge and its responsible application in personal and social contexts (Tenreiro-Vieira and Vieira 2022). A scientifically literate individual applies scientific thinking for both personal and social purposes, integrating knowledge, values, and personal experience to make informed and critical decisions about socioscientific issues (Sadler et al. 2002).

In the context of STS education, the framework of creative critical thinking (CCT) has been proposed. It is conceived as an ethical and effective mode of thinking aimed at generating and evaluating ideas, solving problems, and making responsible decisions (Tenreiro-Vieira and Vieira 2022). This conceptualisation is the result of over 30 years of research in science education across various educational levels. The inclusion of creativity is supported by the strong interconnection between the two modes of thinking (Paul and Elder 2006).

CCT is structured around four dimensions: capacities; knowledge; norms and criteria; and dispositions, attitudes, and values (Table 1).

**Figure 1:**
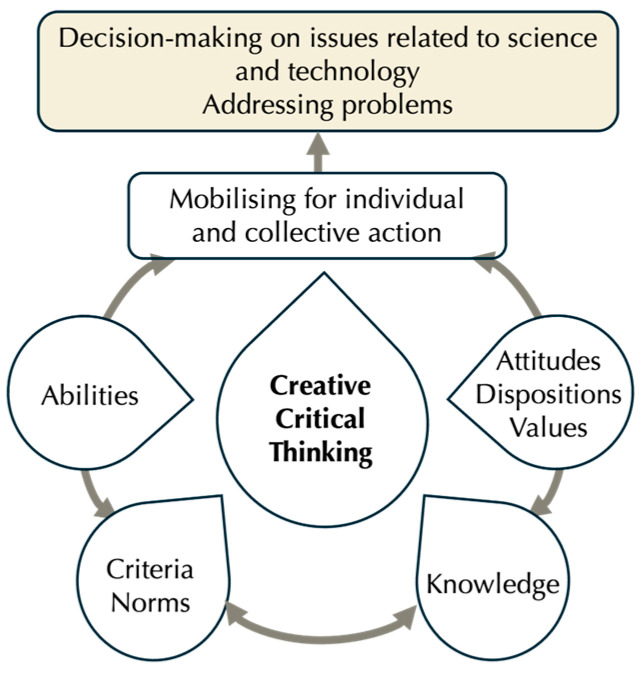
Framework for CCT (adapted from Tenreiro-Vieira and Vieira, 2022).

As shown in Figure 1, CCT is a practical and reflective activity that mobilises complex skills in social interaction, problem-solving, and decision-making related to science and technology.

## 3. Modelling

### 3.1. Modelling as an Epistemic and Reflective Practice

Model-based learning considers models as organising principles of the curriculum, useful for representing, describing, explaining, and predicting phenomena (Gilbert and Boulter 2012). This approach starts from students’ initial ideas, which progressively transform into more explicit, complex, and coherent representations aligned with school scientific models (Oliva 2019). Beyond the learning of models, modelling engages students in their reconstruction, revision, communication, and application to new situations (Justi and Gilbert 2002). This active involvement fosters authentic scientific practices (Gilbert 2004) and the development of modelling competence (Nicolaou and Constantinou 2014). This competence can be developed progressively through instruction and active participation in iterative teaching cycles that resemble the processes of scientific research. In fact, constructing models is a core activity in scientific investigation, and their reconstruction in the classroom can be seen as a form of inquiry. Consequently, many educational proposals are framed in terms of both modelling and inquiry, as these two practices are deeply interconnected and difficult to separate.

Although modelling cycles may vary, they tend to share common phases (Justi and Gilbert 2002; Oliva 2019). According to Justi (2006), the modelling cycle:

- Begins with the elicitation of the purpose of a new model and the formulation of a contextualised problem.
- Continues with the collecting of information, the establishment of an analogy or mathematical tool, and the selection of appropriate codes and representational languages, which enables the formulation of an initial model.
- The model is then tested through empirical or thought experiments to gather evidence. Depending on the results, the initial model may be revised or rejected. When multiple models emerge, they are compared.
- Finally, the scope and limitations of the model are evaluated, it is communicated, and, once consensus is reached, it is applied to problem-solving in various contexts.

Modelling in the classroom must be flexible, adapting to both the context and the characteristics of the students. It may involve only certain phases of the cycle or alter the usual sequence of modelling. In this regard, Gilbert and Justi (2016) argue that modelling cycles do not follow a linear order, as their phases often overlap and involve cognitive processes that are repeatedly engaged, such as analogical reasoning, pictorial representation, thought experiments, and argumentation.

Maurines (2024) builds on this proposal to describe the creative process of modelling as the generation of new and relevant ideas. The macro-processes of creativity are linked to the phases of modelling, while the micro-processes (analogical reasoning, pictorial representations, or thought experiments) emerge through their interactions. These occur alongside argumentation, which plays a central role in both the social dimension of scientific activity and the evaluation of knowledge. This reinforces the idea that involving students in modelling practices can promote the development of their creativity and critical thinking.

Modelling is frequently aligned with the objectives of science education outlined by Hodson (2008), who proposes a comprehensive framework for scientific literacy. This framework encompasses learning science, learning to do science, learning about science, and using science as responsible citizens. Regarding models, this entails not only learning about them but also acquiring the skills to use them, understanding their nature, and applying them within techno-scientific, socio-scientific, and environmental contexts. As Justi (2006, p. 178) asserts, “model construction is an activity with significant potential to engage students in ‘doing science’, ‘thinking about science’, and ‘developing scientific and critical thinking’.”

From this perspective, students should participate not only in epistemic modelling practices but also in reflective ones. Such reflective engagement fosters a deeper understanding of their own learning processes as well as the nature of scientific models. Moreover, it encourages the application of models in contexts relevant to citizenship.

### 3.2. Modelling Competence

Model-based learning not only promotes the construction of models. It also fosters the development of skills, values, and metacognitive knowledge about the nature of models required to perform this practice, commonly referred to as modelling competence (Nicolaou and Constantinou 2014). Although there is no universally accepted definition, modelling competence is generally understood as a complex system of actions that integrates knowledge, cognitive abilities, attitudes, and other non-cognitive elements necessary for representing complex systems or phenomena and for understanding how science operates (Oliva 2019).

**Figure 2:**
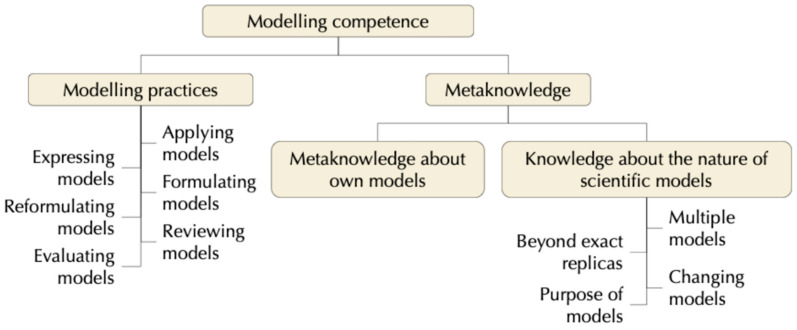
Components of modelling competence. Extended adaptation of the classification by Nicolaou and Constantinou (2014).

Modelling competence primarily includes modelling practices, an integrated set of scientific actions such as expressing, applying, evaluating, revising, formulating, and reformulating models, as well as their use in various contexts (Justi and Gilbert 2002). Additionally, it involves metacognitive reflection about modelling practices, acknowledging that competence extends beyond the mere use of models to include a reflective awareness of their functions, limitations, and purposes (Figure 2).

This metacognitive dimension comprises two components. The first concerns metacognition about one’s own models, involving awareness and self-regulation of cognitive processes during modelling (Nicolaou and Constantinou 2014). The second relates to metacognition about scientific models—reflecting on their nature, functions, and limitations—which promotes an epistemological understanding of models as representations used to explain and predict phenomena, and which may be validated or revised in light of new evidence (Schwarz et al. 2009; Krell and Krüger 2015).

Existing research suggests that these components are interdependent, meaning that learning models, working with them, and understanding their nature are interconnected processes that mutually reinforce one another (Oliva 2019).

## 4. Creative and Critical Thinking, and Modelling-Based Learning

As previously noted, modelling engages students in scientific practices that enable them to critically examine the world around them, construct and evaluate scientific knowledge, develop explanations, make predictions, reflect on the nature of these processes, and apply such knowledge in scientific, technological, and social contexts (Justi 2006). In this regard, integrating practices associated with CCT can enhance the development of modelling competence, given the synergy between scientific thinking and critical thinking (García-Carmona 2023).

Despite this convergence, it is important to explicitly clarify the shared aspects of both approaches. Doing so facilitates the design of teaching proposals that foster their common abilities and enables future research to identify indicators for analysing interactions between modelling and CCT.

Several elements can strengthen both modelling and CCT. On the one hand, the tools and resources employed in modelling processes are fundamental. Among these, analogies play a key role in constructing and reconstructing knowledge, as well as in cultivating the skills, attitudes, and values associated with modelling (Justi 2015; Oliva 2004). Moreover, analogies stimulate evaluative processes (Justi 2015), which are central to critical thinking, while also encouraging imagination and creativity (Olguín 2021). Similarly, elements such as scale models, mock-ups, and thought experiments can significantly contribute to model construction and evaluation.

On the other hand, argumentation—when considered through the lens of modelling—is closely linked to other core components of CCT, including communication and information management, which are highly interconnected processes. Additionally, the exploration of socioscientific issues (SSI) offers diverse scenarios for practising argumentation within the context of modelling (Durak and Topçu 2021).

### 4.1. Analogies and Resources for Modelling

The construction or reconstruction of school scientific models entails a creative and critical endeavour. It involves analysing and simplifying reality, as well as developing skills for the (re)construction, use, revision, and reformulation of models (Justi and Gilbert 2002; Justi 2006). Analogies are a particularly powerful tool for model (re)elaboration in educational contexts (Yerrick et al. 2003), as they make scientific concepts more accessible by linking them to familiar and less abstract referents. In addition, they foster processes, skills, and epistemological perspectives that are central to both science and modelling practices (Oliva 2004). These elements are encompassed in the term analogical reasoning, which is not only relevant when working with analogies but also represents a valuable cognitive skill to be cultivated in science education.

Analogical reasoning involves various processes, including communicating analogies by establishing appropriate relational correspondences, applying analogies to interpret reality, making predictions, conducting thought experiments, evaluating and reconstructing analogies, and independently generating new ones. The associated skills include selecting a suitable analogue, mapping analogical relations, making inferences to characterise the target concept, critically evaluating the analogy, and generalising inferences (Mozzer and Justi 2018). Furthermore, students must develop an epistemological understanding of what analogies are, how they are used, and how to evaluate their utility.

To develop analogical reasoning, it is necessary to design activities that actively engage students in the elaboration and development of analogies (Ramos et al. 2021), while promoting a dialogic learning environment that enriches the process. Such activities should follow a clear and coherent structure aligned with the intended educational goals. Two models that support this approach are Glynn’s (1991) Teaching with Analogy (TWA) model and the analogical modelling framework proposed by Mozzer and Justi (2018), the latter integrating analogies within the broader modelling cycle.

Both models share a procedural structure that enables the meaningful integration of analogies in the learning of complex phenomena. By analysing their common features, a shared framework can be articulated, consisting of the following stages:

- a contextualisation phase, in which the target phenomenon or object is introduced;
- a generation phase, where a suitable analogy is proposed or constructed by selecting an analogue domain that facilitates the establishment of relevant relations;
- a correspondence phase, aimed at making explicit the similarities and differences between the analogue and the object, thus enhancing the understanding of their structural relationships;
- an application phase, in which the analogy serves as a tool for formulating explanations, hypotheses, or representational models;
- an evaluation and revision phase, involving critical reflection on the limitations, scope, and potential reformulations of the analogy or the model derived from it.

In addition to activities focused on the generation or reconstruction of analogies, it is also important to include those centred on their application for hypothesis formulation or problem-solving, as well as activities that promote metacognition regarding analogy-based learning and critical reflection on the nature of science. Recognising the limitations of analogies contributes to understanding the provisional and approximate nature of scientific models, discouraging their perception as absolute representations of reality. This, in turn, supports a more humanised and less dogmatic view of scientific knowledge (Heywood 2002).

The above suggests a close relationship between analogical thinking and CCT, supported by studies showing improvements in students’ critical thinking after working with analogies (Kapetanakis et al. 2024; Suryanda et al. 2020; Bulegon and Tarouco 2015; Herranz-Hernández et al. 2024).

Firstly, analogical thinking activates procedural skills common to critical thinking, such as analysis and reasoned evaluation (Scriven and Paul 2007), facilitating the conceptualisation, comparison, and application of models. For example, Suryanda et al. (2020) conclude that learning through analogies improves the identification and explanation of problems and the evaluation of assumptions, while Kapetanakis et al. (2024) find that students show greater flexibility in revising their answers.

Secondly, the development of analogical reasoning can foster critical dispositions, attitudes, and values necessary for modelling, such as valuing one’s own ideas and maintaining an open mind to modify previous conceptions (Oliva 2004).

Thirdly, creativity, which is interconnected with critical thinking (Paul and Elder 2006), is enhanced through analogical reasoning, which, together with argumentation, pictorial representations, and communication, constitutes the creative microprocesses of modelling (Maurines 2024; Nersessian 2002).

Fourthly, working with analogies brings students closer to authentic science, promoting citizen participation in technoscientific decisions (Acevedo Díaz et al. 2005). Historically, analogies have facilitated conceptual change (Nersessian 2002), such as in fluid mechanics (Ulazia Manterola 2015), electromagnetism (Acevedo-Díaz 2004), and the interpretation of celestial orbital motion (Oliva and Acevedo-Díaz 2011). The analysis of historical cases improves understanding of the nature of science and fosters positive attitudes by portraying scientific reasoning as accessible, valuing the logic and rationality of scientific theories over pseudoscience, and acknowledging the limitations of both scientific models and science itself.

Fifthly, analogies contribute to conceptual learning and the development of complex conceptual networks, which are fundamental to critical thinking, since they allow for establishing relationships, evaluating the coherence of arguments, and transferring knowledge to other contexts. Furthermore, they promote metacognition about scientific practice (Brown and Salter 2010), which is essential for engaging with social issues.

Sixthly, constructing analogies that can be used as justifications, as part of arguments, or to support them is one of the skills required for developing arguments in the scientific context. In the same context, the process of counter-argumentation may require identifying limitations in analogies to justify an argument or in the relations established between these analogies and the original claim (Justi 2015).

In sum, the foregoing analysis suggests that analogies can contribute not only to conceptual learning and modelling but also to the development of skills and dispositions associated with CCT.

Besides analogies, other common resources in modelling, such as thought experiments or pictorial representations in any of their formats, including scale models and mock-ups, play a fundamental role in the generation of scientific knowledge (Nersessian 2002). Both can activate creative processes and involve reflective acts that enable the review and evaluation of the models being worked on. The history of science offers numerous examples. For instance, analogy, mental pictorial representation, and simulation as a thought experiment were tools employed by Maxwell in developing his theory of the electromagnetic field (Nersessian 2009); the construction of molecular-scale models was essential for Watson and Crick in the elaboration and revision of the double helix DNA model (Acevedo-Díaz and García-Carmona 2017).

### 4.2. Argumentation from the Perspective of Modelling

Argumentation can be considered the epistemological core of scientific and socioscientific thinking, closely linked to modelling, critical thinking, and the formation of an active citizenship (Jiménez-Aleixandre and Puig 2011; Vázquez-Alonso and Manassero-Mas 2018b). Its relevance is heightened in scientific communication and in modelling-based teaching that adopts a socioconstructivist approach, where interactions between students and teachers guide the entire process (Boettcher and Meisert 2011).

Although argumentation extends beyond a dialogic process involving internal cognitive processes and the production of artefacts, it remains a fundamental component of human communication and, in particular, of scientific communication (Boettcher and Meisert 2011). In modelling-based teaching, communication plays a central role in classroom discourse, aligning with the socioconstructivist framework. Interactions among students and between students and teachers set the pace for modelling processes. The communicative acts involved in modelling frequently entail argumentative reasoning for justifying ideas and persuading others of their validity, requiring scientific language appropriate to the argumentation. However, expressing models can be challenging not only because it involves mastering new vocabulary and argumentative structures but also because scientific language employs multiple complex codes. Moreover, promoting social interaction and communication is essential for generating a diverse and imaginative environment that stimulates creativity (Fleissner-Martin et al. 2024), which is particularly important in modelling.

Argumentation is also closely tied to information management; indeed, it promotes skills for handling information (Jiménez-Aleixandre and Puig 2011). Information management is understood as the capacity to comprehend, critically analyse, interpret, and synthesise information. The search for information from reliable sources, along with its management, is important for applying critical thinking to decision-making or problem-solving, both in socioscientific and purely scientific contexts (García-Carmona 2023). Similarly, the search for and management of information are very present in the modelling process in the classroom, both in the initial phase, where information is needed to develop a first model, and in the revision and evaluation of the models generated, requiring, for example, evidence often extracted from diverse sources (Justi 2015).

CCT is fundamental for the production, evaluation, and use of arguments, with argumentation being one of its most relevant components (Jiménez-Aleixandre and Puig 2011; Saiz and Rivas 2023). Like CCT, modelling involves reviewing and evaluating ideas to construct a creative product—a model that addresses problems or supports decision-making. This parallel underscores the close synergy between argumentation and modelling (Evagorou et al. 2020).

Building on the purposes of science education (Hodson 2008)—learning science, learning to do science, learning about science, and learning to use science in citizenship—Boettcher and Meisert (2011) propose a model for argumentation grounded in modelling-based learning. From this perspective, argumentation comprises four dimensions: knowledge of models and evidence; the ability to argue; knowledge about arguments and argumentation; and the use of argumentation to exercise citizenship. This final dimension represents an extension to Boettcher and Meisert’s original framework.

The first dimension, knowledge of models and evidence, is considered the foundation of argumentation. The better a model and its supporting evidence are understood, the stronger the arguments that can be built to support its plausibility. However, knowledge about models and evidence can vary and evolve significantly throughout the argumentation process (Boettcher and Meisert 2011).

The second dimension concerns the ability to argue, which takes on different characteristics depending on whether the issue is scientific or influenced by social or personal factors. In scientific contexts, argumentation involves constructing well-supported claims—whether empirical, bibliographic, or analogical—managing evidence, considering counterarguments (i.e., examining conditions that could falsify a claim through diverse perspectives), developing alternative models, and engaging in refutation, which entails defending one model or idea against another (Justi 2015). These practices aim to construct meaning, articulate ideas, and, in some cases, persuade.

The third dimension acknowledges the importance of metacognition about argumentation. Involving students in modelling practices requires them to understand what argumentation is, how arguments are constructed, and the role argumentation plays in modelling (Passmore and Svoboda 2012). This metacognitive understanding can be enriched by recognising the implicit processes involved in searching for and managing information. This includes being aware of source reliability criteria and the communicative dynamics associated with argumentation.

The fourth dimension, using argumentation to exercise citizenship, refers to the ability to engage in argumentative discourse in socioscientific contexts, where various social dimensions—political, economic, cultural, ethical, or religious—as well as personal aspects such as emotions, interact with scientific considerations. Particularly relevant in such contexts are information management skills and communication competence.

This entails the development of specific skills, the acquisition of dispositions, attitudes, and values, as well as the adoption of standards for assessing the quality of thinking and value judgements, in line with the dimensions of critical thinking (Tenreiro-Vieira and Vieira 2022). Similarly, Jiménez-Aleixandre and Puig (2022) point out that argumentation in socioscientific contexts is shaped by the development of independent opinions and a sense of social justice. Taking these considerations into account, socioscientific argumentation is influenced by multiple factors that make it complex, yet also engaging and essential from the perspective of critical thinking development. Working with socioscientific arguments in the classroom does not mean replacing scientific arguments, but rather that both types of reasoning should coexist, just as they do in society and even within the scientific community.

First and foremost, it is important to promote respect for scientific evidence (García-Carmona 2023), particularly that which comes from recognised institutions. This does not mean that evidence is beyond discussion, but rather that scientific knowledge is consensual and as objective as possible and cannot be replaced by denialist or pseudoscientific messages.

Secondly, a balance between different types of arguments must be sought, fostering a critical mindset that enables students to take responsible positions and contribute to solutions for SSI (Kolstø 2001; Sadler 2004). Although emotional criteria are necessary—given the complexity and social impact of many SSI—people often adopt positions based on emotional intuitions (Haidt 2012) rather than on rational arguments. However, in matters where social justice or sustainability criteria must prevail, it is legitimate to dismiss arguments that seek personal benefit.

Thirdly, arguing in the classroom about SSI requires teacher guidance to steer the discussion, ensuring that students base their opinions on evidence without prescribing a single correct answer. This involves helping students identify trustworthy sources, distinguish facts from perceptions, and explore the issue from multiple perspectives, thus allowing different solutions to emerge.

Regarding the implementation of this dimension in the classroom, teaching proposals that integrate SSI into modelling processes provide an appropriate framework for the development of socioscientific argumentation (Sadler 2004). As Marchán-Carvajal and Sanmartí (2014, p. 703) point out: “SSI introduces a significant axiological component grounded in critical thinking and oriented towards socially responsible decision-making, goals that education in context cannot overlook.” In fact, modelling can play a key role among teaching approaches aligned with the objectives of scientific literacy and critical thinking (Durak and Topçu 2021; Schwarz et al. 2009). This approach provides students with tools to understand science, evaluate information, consider diverse perspectives, and apply knowledge to socially relevant contexts, enabling them to make informed decisions and address problems related to science and its social impact (Durak and Topçu 2021). Therefore, it can be said that critical thinking contributes to strengthening students’ modelling competence.

It is important to note that there is a gradient in how SSI is integrated into modelling. In some cases, only scientific aspects are addressed. However, as economic, political, cultural, ethical, religious, or emotional dimensions are incorporated, a broader spectrum emerges (Jiménez-Aleixandre and Puig 2011). A proposal’s position along this spectrum may depend on factors such as the official curriculum, its adaptation to students’ context, or the nature of the scientific topic being explored, considering the sociological dimension of science (Acevedo-Díaz and García-Carmona 2016).

**Figure 3:**
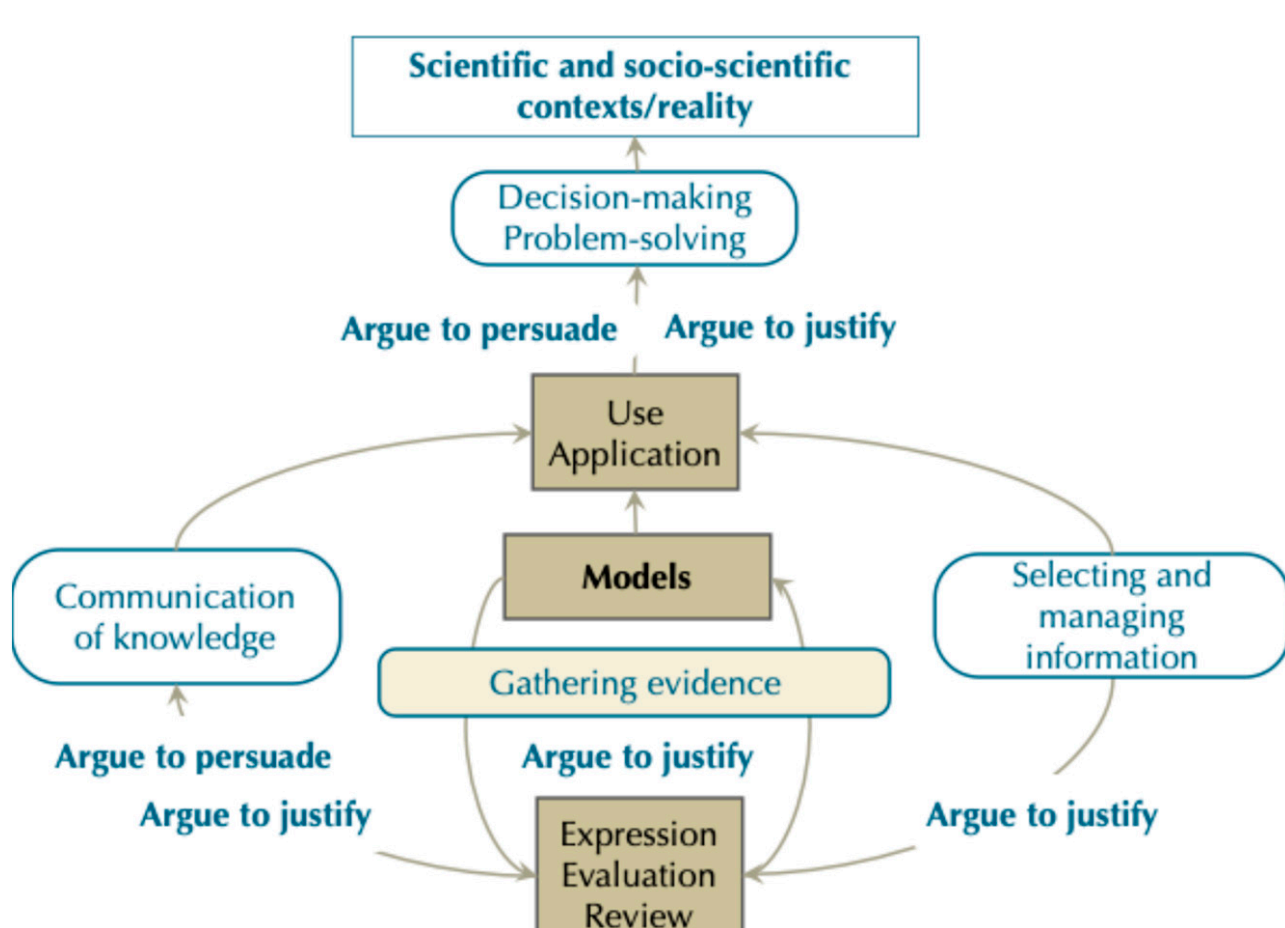
The role of argumentation, information management, and communication within the modelling process.

The design of modelling-based teaching proposals aimed at developing CCT, and which include SSI to a greater or lesser extent, should explicitly and reflectively integrate argumentation, information management, and communication within the modelling processes, while considering the four dimensions. Research in science education provides examples of teaching proposals that, to varying degrees, incorporate these features (Evagorou et al. 2020; Jia and Ren 2025; Ke et al. 2020). Figure 3 guides by outlining the appropriate moments for this integration, highlighting the central role of argumentation in modelling, the stages that require information gathering and management, and the most relevant communicative acts.

The role of argumentation in modelling, highlighted in Figure 3, has been described by several authors (Gilbert and Justi 2016). Boettcher and Meisert (2011) emphasise its importance in the evaluation of models—whether to discard, modify, establish more appropriate ones, or compare them. Passmore and Svoboda (2012) broaden this perspective by situating argumentation across all stages of the modelling process. Extending this perspective, Justi (2015) and Martins (2020) provide evidence, through educational experiences, that students demonstrate argumentative skills when handling evidence, evaluating models, planning experiments, interpreting data, justifying models, comparing and applying them, making predictions, and engaging in decision-making. Conversely, argumentative skills also support and enhance modelling processes (Justi 2015). In addition, modelling requires justification of what information is selected and how it is used. Argumentation is therefore necessary not only to justify ideas but also to persuade others of their validity, particularly when communicating the models developed and when proposing solutions or defending positions related to SSI.

Communicative acts are constantly present throughout modelling processes. The initial expression of models, as well as the interactions that occur during their revision and evaluation, support progression from initial, implicit, and personal mental models to more explicit, complex, and coherent representations aligned with the school’s scientific model. Similarly, the communication of the final model contributes both to improved expression and to the appropriation of the model by students (Justi 2006; Ageitos Prego and Blanca 2016).

Modelling, as a school-based scientific practice, provides an opportunity to evaluate knowledge and justify confidence in it, which develops alongside the learning process (Fischer and Huff 2025). This metacognitive reflection can support reasoning in controversial scientific contexts. Considering the limits of one’s own knowledge encourages the search for information beyond prior beliefs, facilitates the integration of new evidence into judgments, and provides criteria for distinguishing accurate information from misinformation, promoting flexibility in initial positions (Fischer and Fleming 2024; Fischer and Huff 2025). Argumentation-centred scientific communication plays a crucial role in fostering metacognitive accuracy, helping individuals cultivate justified confidence in knowledge and supporting informed beliefs and decision-making (Fischer and Huff 2025).

## 5. Implications and Future Perspectives

This study proposes potential points of intersection between two long-standing educational frameworks—the development of CCT and modelling-based learning. Although further research is required to validate these convergences.

First, modelling resources, especially analogies, stand out due to their numerous points of connection with the development of CCT: (1) they activate analytical and evaluative reasoning skills; (2) they foster a tendency to act critically and to acquire the attitudes and values necessary for modelling; (3) they enhance creativity; (4) they improve attitudes towards science; (5) they support the understanding of scientific concepts and the nature of science; and (6) they strengthen argumentative skills, since analogies involve a form of reasoning. For analogies to be effective, they must be used following certain guidelines. Two particularly useful models in this regard are Glynn’s TWA model (1991) and analogical modelling (Mozzer and Justi 2018). Although less explored in this study, pictorial representations and thought experiments play a similar role to analogies, and further research is needed to delve deeper into the relationship between modelling and critical thinking.

Second, argumentation is closely connected to other essential CCT-related skills such as communication and information management. To integrate them into modelling practice, four dimensions can be considered, expanding on Boettcher and Meisert’s (2011) framework on argumentation: (1) the importance of model and evidence knowledge as a foundation for argumentation and communication, and how developing these capacities supports students’ model progression; (2) the learning of these skills as processes situated within the context of science; (3) the development of metacognitive knowledge related to the three skills; and (4) citizenship engagement through argumentation, information management, and communication. This last dimension can be addressed by integrating SSI into modelling-based learning, which appears particularly appropriate for the development of CCT. Similarly, intentionally incorporating CCT seems to support students’ modelling competence. To facilitate future research on the design of teaching proposals focused on modelling and CCT development, this study identifies specific moments within the modelling process where these skills are activated.

This article is framed as a perspective piece that seeks to contribute to the challenge of intentionally and explicitly integrating the development of CCT into modelling-based scientific practices in school settings. Its purpose is to open a line of theoretical reflection and to lay the groundwork for future research in this area, rather than to provide a systematic model or a set of ready-to-use teaching resources. In this sense, the paper offers an initial framework that highlights the potential for systematically incorporating CCT into concrete teaching proposals while recognising the gap between the pressing need to develop CCT in citizens and the current classroom reality. Further progress must be pursued through empirical research, the design of teacher scaffolding tools, some of which are suggested in this work, and the development of assessment processes, which have not been addressed here due to space limitations. These advances should lead to concrete teaching proposals, whose effectiveness will need to be evaluated against their design intentions.

For teachers to adopt this type of proposal, it is essential to focus on teacher education, both initial and ongoing, across all educational levels, since CCT can be fostered from an early age (Tenreiro-Vieira and Vieira 2022) and should be developed throughout life.

In conclusion, and in response to the hypothesis set out in the introduction, this study supports the idea that there is a synergy between the development of CCT and modelling. Modelling practices that incorporate resources such as analogies—as well as pictorial representations and thought experiments—not only enhance students’ modelling competence but also promote their skills in argumentation within socioscientific contexts, communication, and information management. These practices can, in turn, provide the tools needed to exercise CCT in socially relevant issues with a scientific component and to act as engaged and responsible citizens.

## Figures and Tables

**Table 1:** Dimensions of CCT (adapted from Tenreiro-Vieira and Vieira, 2022).

Dimension	Description	Elements
Abilities	Cognitive skills (analytical, evaluative, and creative) are necessary to think effectively and make rational decisions.	- Basic clarification: summarizing, identifying or providing reasons, arguing, communicating. - Basic support: evaluating the credibility and reliability of sources, observations, etc. - Elaborate clarification: operationally defining, constructing taxonomies or categories, making comparisons and contrasts, identifying patterns and relationships. - Inferences: making and evaluating inductions, value judgments. - Strategies and tactics: deciding on an action, interacting with others. - Creativity: originality, flexibility, fluency, elaboration. - Innovation and executive functions: conflict resolution, assertive communication, negotiation.
Dispositions, attitudes, and values	Tendency to act critically and creatively (critical spirit).	- Open-mindedness and tolerance for uncertainty. - Active engagement, trust, and participation. - Seeking and providing reasons and assuming them publicly. - Using and citing reliable sources and striving to stay well-informed. - Being sensitive to others’ feelings, knowledge levels, and degree of elaboration. - Collaborating with integrity and intellectual satisfaction. - Respecting evidence, perseverance, resilience, and adaptability. - Values such as honesty and intellectual humility.
Norms or criteria	Standards to evaluate the quality of thinking and value judgments.	- Clarity and rigor. - Precision, considering the situation. - Consistency and coherence. - Systematicity. - Intellectual independence. - Prudence and consultation. - Impartiality and ethics. - Civic initiative. - Self-regulation and metacognition. - Planning and strategy.
Knowledge	Scientific and technological knowledge and understanding of critical thinking itself.	- Big ideas and core concepts in science and technology. - Scientific and technological theories and explanations. - History of science. - Nature of science. - Current fields and research contexts in science and technology. - Understanding of creative critical thinking and its components.

## Data Availability

No new data were created or analysed in this study. Data sharing is not applicable to this article.
